# Successful Management of Whipple’s Disease in a 46-Year-Old British Woman: A Case Report

**DOI:** 10.7759/cureus.77333

**Published:** 2025-01-12

**Authors:** Gayathri Jayakumar, Vikash S Sagar

**Affiliations:** 1 Gastroenterology, Frimley Park Hospital, Camberley, GBR

**Keywords:** diarrhea, esophagogastroduodenoscopy, gastrointestinal tract, periodic acid-schiff, tropheryma whipplei, weight loss, whipple’s disease

## Abstract

Whipple's disease (WD) is a rare systemic infection, notoriously difficult to diagnose due to the presence of common, ambiguous, or constitutional clinical characteristics. We present the case of a 46-year-old British woman, who presented to the emergency department (ED) of Frimley Park Hospital, United Kingdom, with chief complaints of progressive weight loss, abdominal pain in the right upper quadrant, diarrhea bloating, and melena or rectal bleeding that had been ongoing for five months. Baseline studies were conducted outside of our hospital. The patient's secondary pathology included pulmonary sarcoidosis (PS), pulmonary hypertension (PH), pulmonary artery aneurysm (PAA), hypothyroidism (HT), and learning impairments. After evaluating all of the medical reports, an esophagogastroduodenoscopy (OGD) revealed the presence of oedematous mucosa, and a biopsy from the second portion of the duodenum confirmed the diagnosis of WD. *"Tropheryma whipplei" *was not detected using polymerase chain reaction (PCR), periodic acid-schiff (PAS), or fungal staining. As a result, early diagnosis should be a top priority for such individuals, with OGD and biopsy serving as the primary diagnostic tool. Furthermore, ongoing follow-up with the patient is required, as recurrence is common and can occur even after a full course of antibiotic treatment. This case report underlines that, due to the constitutional characteristics of the clinical manifestations, decision-making should be based on current clinical features as well as any related secondary diseases, rather than only signs and symptoms.

## Introduction

Whipple's disease (WD), coined by Nobel laureate George Hoyt Whipple (1907), was initially termed intestinal lipodystrophy owing to lymph space accumulation of neutral fats and fatty acids [1]. Whipple described WD as characterized by a progressive loss of weight and strength, neutral fat and fatty acid-dominated feces, vague gastrointestinal symptoms (malabsorption), and an unusual form of arthritis [2]. *"Tropheryma whipplei" *(TW), a gram-positive bacillus that normally occurs in soil and belongs to the diverse class Actinomycetales, is the cause of WD. This uncommon, persistent, and infectious systemic disease mostly affects the gastrointestinal system [3]. Only 1,000 occurrences of WD have been documented globally [4], with a yearly rate of three in a million, and if left undiagnosed and untreated, WD can be lethal [5,6]. Clinically, there are two stages: (1) starting or prodromal, and (2) stagnant or latent. The initial level is marked by joint compromise. The subsequent stage is distinguished by localized symptoms, diarrhea, and weight loss, depending on the impaired organ. WD recurrences may arise in as much as 30% of cases, usually detrimental to their neurological systems.

According to reports, WD requires an average of seven years from the individual's first manifestations to the disease's detection, due to which the disease could be fatal or exhibit some severe aftereffects throughout this time [7]. TW is naturally occurring and can be found in the stool specimens (1% to 11%) of healthy people, and it has also been projected that a fraction of one percent (0.01%) of those infected with TW will acquire WD [8], and vulnerability is determined by host genetics and immune system responses. TW cultures can only be obtained in specialized testing facilities, making diagnosis challenging [9]. The most common method of diagnosis is a duodenal biopsy, which showed that periodic acid-schiff (PAS) positive staining foamy macrophages (lamina propria), yet polymerase chain reaction (PCR) analysis has developed as a more convenient option with excellent accuracy and specificity [10]. WD is difficult to diagnose because it is an infrequent disease, and vague or localized clinical manifestations depending on the organ involved often make it prone to being consistently misinterpreted and underdiagnosed. Hence, the present case report demonstrates a WD case in a 46-year-old who presented with constitutional clinical features and has been identified to be associated with pulmonary sarcoidosis (PS), pulmonary hypertension (PH), pulmonary artery aneurysm (PAA), hypothyroidism (HT), and learning difficulties (LD).

## Case presentation

A 46-year-old British woman, without any history of travel to tropical countries and a non-smoker, presented to the emergency department (ED) of Frimley Park Hospital, United Kingdom, in February 2022, with chief complaints of progressive weight loss, abdominal pain in the right upper quadrant, diarrhea, bloating, and melena or rectal bleeding, which had been ongoing for five months, following a campylobacter infection.

Her health issues commenced at the onset of the COVID-19 pandemic in 2020. She was initially investigated by the hematology team for suspected lymphoma due to her unexplained weight loss. However, abnormally elevated brain natriuretic peptide (BNP) and a chest radiograph revealed diffuse interstitial nodular opacities throughout the lungs bilaterally, along with bilateral perihilar consolidations, led to the diagnosis of PAA, PH, and PS. Various tests, including complete blood count, QuantiFERON (for tuberculosis), an autoimmune screen for spinal muscular atrophy, and tissue transglutaminase IgA (for celiac disease), returned normal or negative results. In February 2022, the patient presented to our hospital with a five-month history of persistent diarrhea and reported that she was recently diagnosed with campylobacter and sought treatment outside our hospital. Her initial fecal PCR test was positive, but the most recent test returned negative. She does not know the specifics of the antibiotic treatment she received for this infection at the other hospital. The family medical history revealed that her aunt died of bowel cancer at the age of 62.

**Figure 1:**
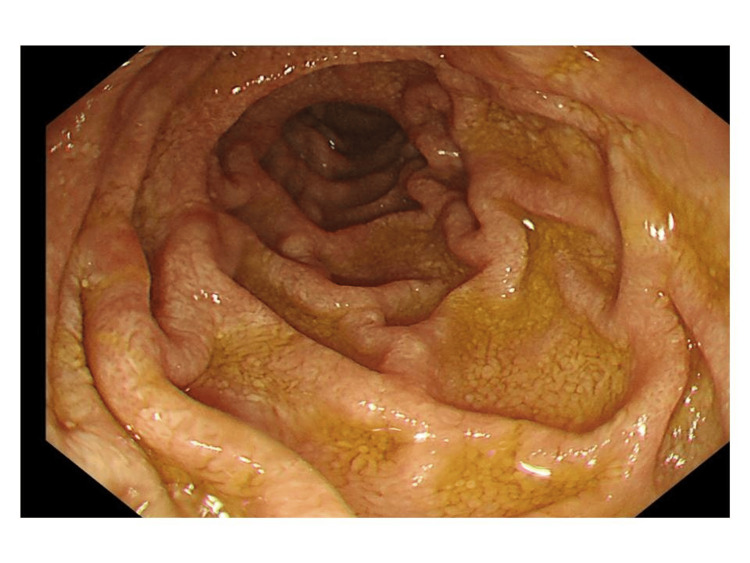
Endoscopic image of the second part of the duodenum showing oedematous mucosa.

**Figure 2:**
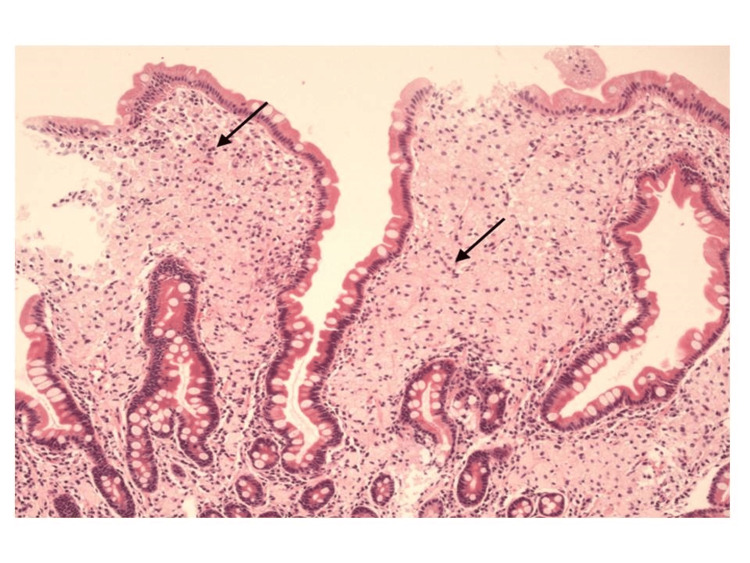
Duodenal lamina propria demonstrating infiltration by foamy macrophages (arrows), shown with H&E staining.

**Figure 3:**
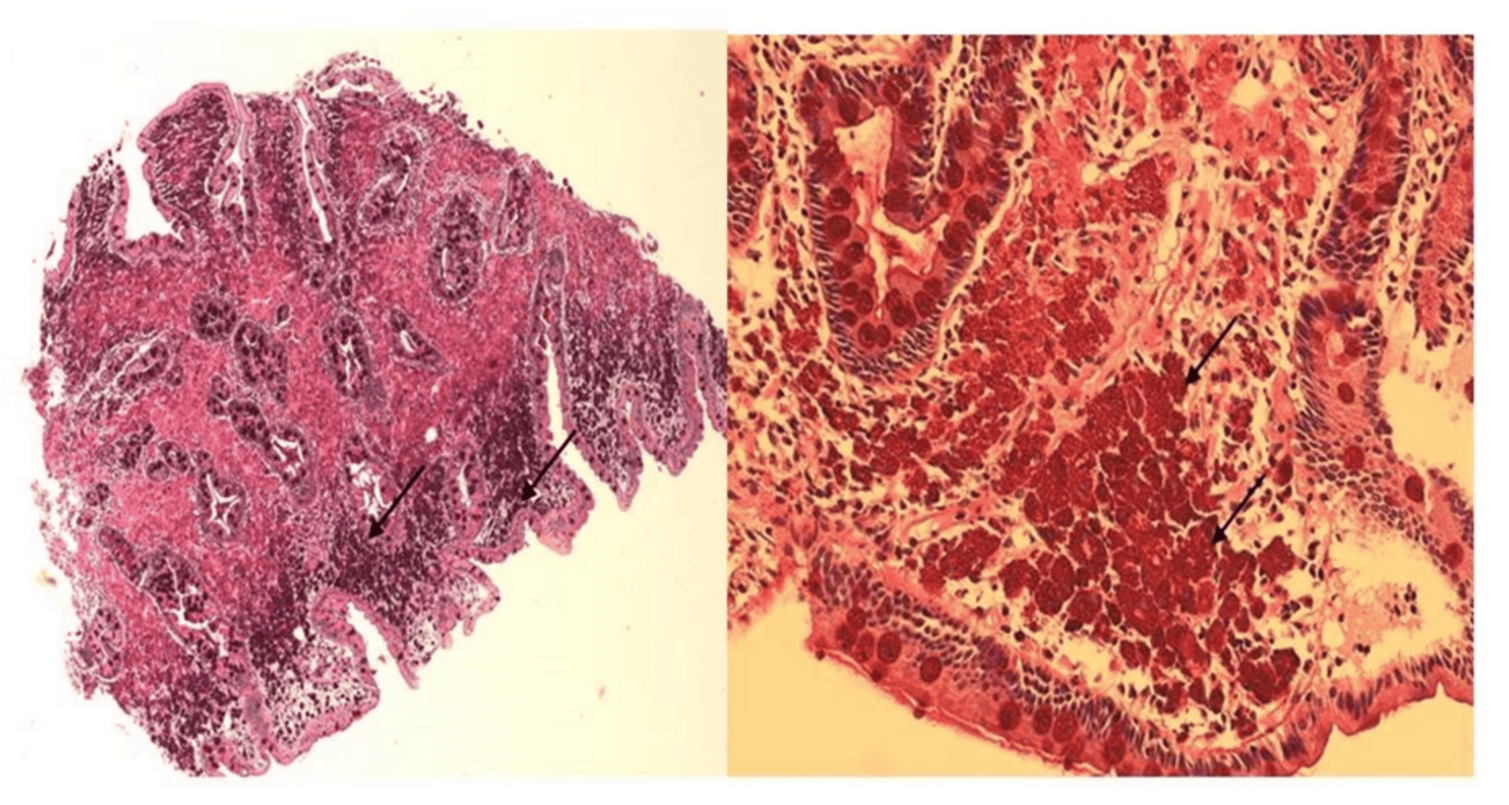
Depiction of characteristic D-PAS positive granular staining within the macrophages (arrows). D-PAS refers to Periodic Acid-Schiff (PAS) with diastase.

**Figure 4:**
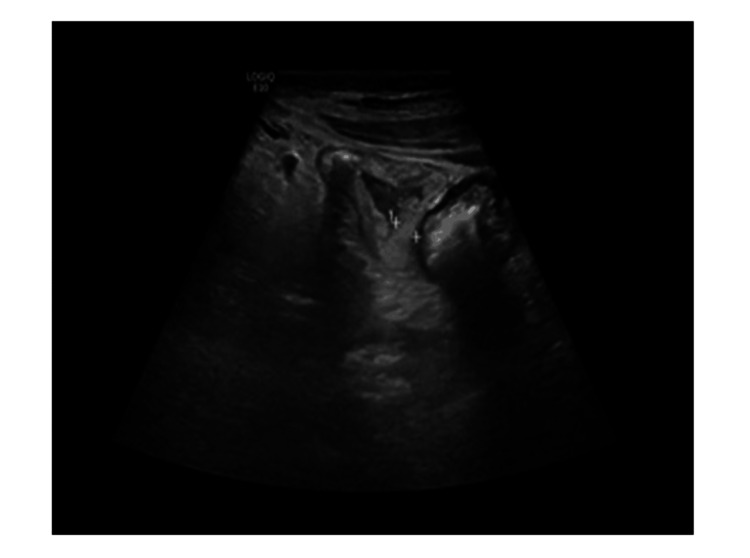
Ultrasound image of the small bowel.

**Figure 5:**
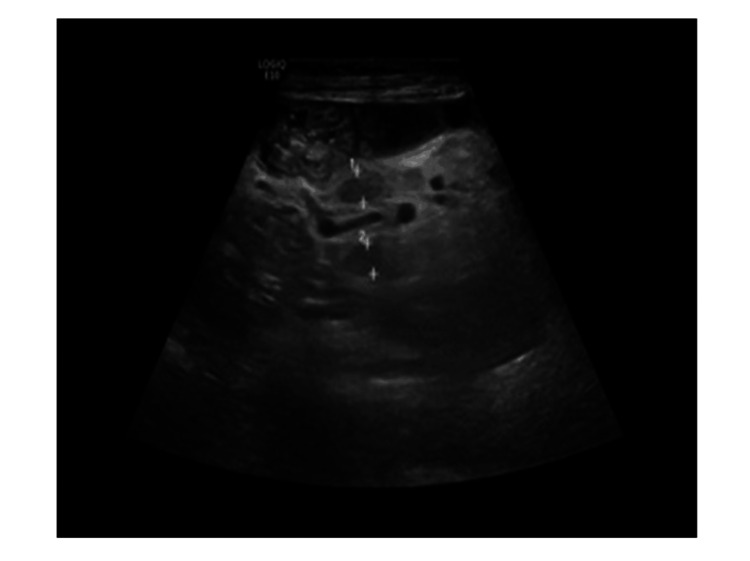
Ultrasound image showing an expanded jejunum with hyperechoic mesentery and multiple reactive lymph nodes.

**Figure 6:**
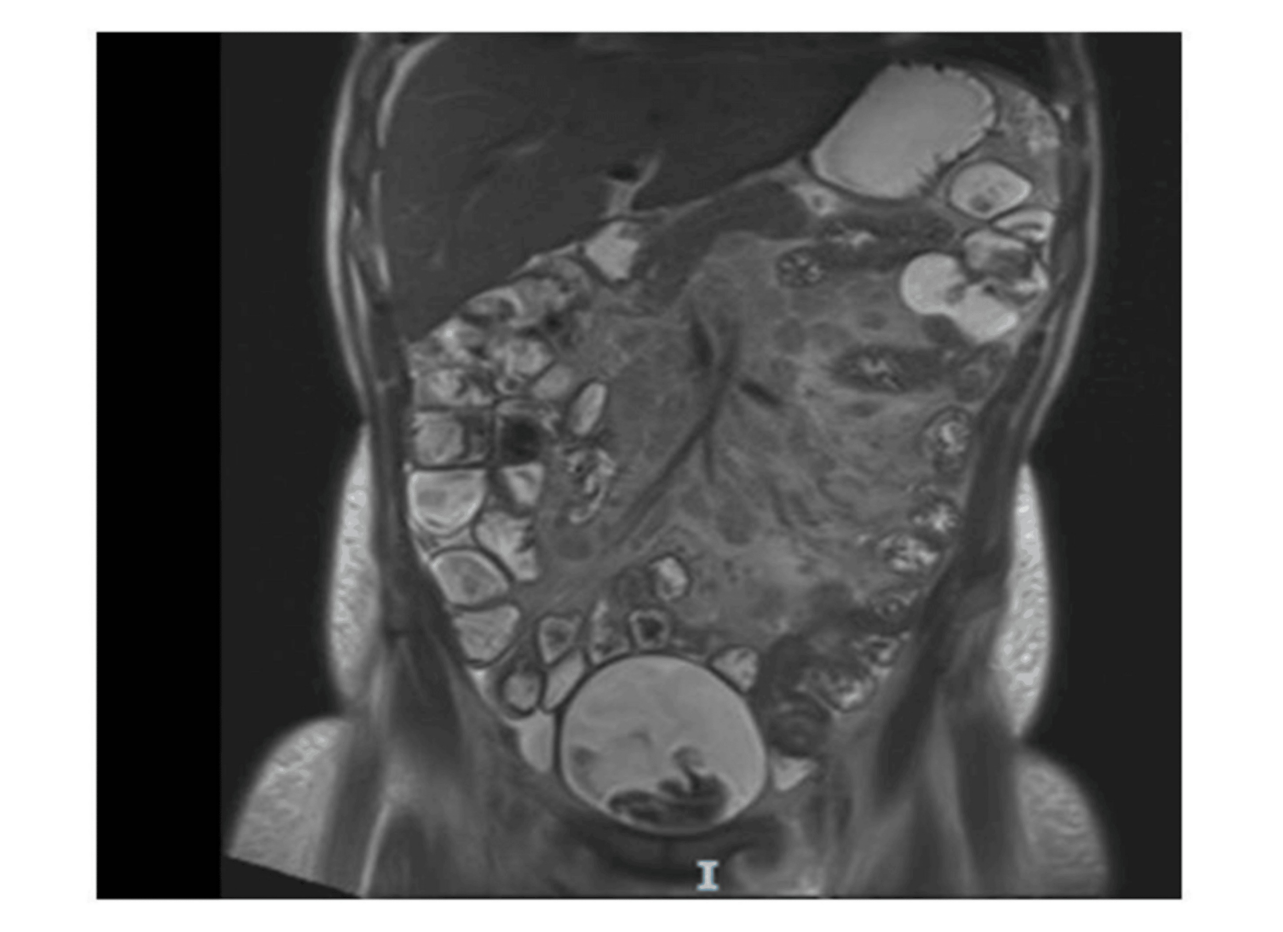
MRI image demonstrating a mesenteric nodal mass and extensive lymphadenopathy. MRI: Magnetic resonance imaging

During a physical examination in our ED, it was confirmed that she had lost 15 kg of weight over the past three weeks. With a family medical history of bowel cancer, the investigations were aimed at ruling out gastrointestinal malignancies amongst other differential diagnoses. All possible causes for her diarrhea were explored, including gastrointestinal sarcoidosis and iatrogenic, i.e., possible medication effects from omeprazole and alendronate. Due to notably elevated fecal calprotectin, it was decided to investigate inflammatory bowel disease through an MRI of the small intestine and colonoscopy. However, given the patient's deteriorating hemoglobin levels and the risks associated with MRI in the context of PH, this was deferred in favor of an unsedated colonoscopy and abdominal ultrasound small bowel as an outpatient. However, subsequent presentation to the ED, marked by intermittent rectal bleeding, malaise, and worsening symptomatic anemia, prompted admission and inpatient flexible sigmoidoscopy, revealing a healthy colon distally but preventing assessment of the cecum and terminal ileum due to solid stool. Imaging studies, including ultrasound and MRI of the small bowel, unveiled widespread enteric inflammation, particularly affecting the jejunal mesentery and proximal ileum, accompanied by mesenteric lymphadenopathy. An esophagogastroduodenoscopy (OGD) yielded unremarkable macroscopic findings of oedematous-appearing mucosa (Figure 1), and a small biopsy taken from the second portion of the duodenum region was sent for histopathological and PCR analysis. The histopathology report indicated the presence of WD, while PCR results were negative for WD. Hematoxylin and eosin (H&E) staining of duodenal lamina propria showing intrusion of foamy macrophages as shown in Figure 2. Similarly, PAS with diastase (PAS-D) established staining of macrophage cells due to the granules present within the cells (Figure 3). Specimens were found to be negative for fungal growth. Importantly, Ziehl-Neelsen staining was negative, and there was no increase in intraepithelial lymphocytes or gastric metaplasia. Images from a small bowel ultrasound (Figure 4) revealed an expanded jejunum with a hyperechoic mesentery and multiple reactive lymph nodes (Figure 5). Additionally, MRI findings demonstrated a mesenteric nodal mass and extensive lymphadenopathy (Figure 6), providing a comprehensive understanding of the condition.

After consultation with microbiology and multi-disciplinary team discussions, the patient was treated with ceftriaxone 2 grams, intravenously once daily for two weeks, and this was followed by oral co-trimoxazole for one year as secondary prophylaxis, given the potential fatality of untreated WD. The patient responded positively to the antibiotic treatment, completely recovering from diarrhea, which confirmed the presence of WD. After completing the antibiotic course, the patient was followed up regularly to monitor for recurrence. Within a couple of days of starting antibiotic treatment, a significant clinical response in the patient was seen. The patient’s diarrhea had resolved, and biochemical markers also started to show improvement (Tables 1-2). Follow-up clinic at six months following discharge showed continued improvement with complete resolution of gastrointestinal symptoms, and the patient had started regaining weight. Given the patient’s complex comorbidities and overwhelmingly positive response to treatment, the decision was made to not have repeated endoscopic biopsies done for confirmation of histological surveillance or repeat PCR/cultures.

**Table 1:** Investigation outcomes of the reported patient’s case. RBC: Red blood cell count; WBC: White blood cell count; PCR: Polymerase chain reaction

Investigations	Patient value/outcome	Reference range (units)
RBC count	5.1	4.2 to 5.4 million cells/microliter (µL)
WBC count	10,165	4,500-11,000 white cells per microliter (µL) of blood
Platelet count	365,000	157,000 to 371,000 per microliter of blood
Thyroid-stimulating hormone (TSH)	11.3	0.45-4.5 milliunits per liter (mU/L)
QuantiFERON	Negative	-
Serum angiotensin-converting enzyme (ACE)	69	8-52 units per liter (U/L)
Fecal calprotectin	375	50-200 μg/mg
Serum brain natriuretic peptide (BNP) levels	>276.7 pg/mL	Less than 100 picograms per milliliter (pg/mL)
Autoimmune screen of Spinal Muscular Atrophy (SMA)	Negative	-
Tissue Transglutaminase IgA	Negative	-
Lymph node biopsy	Granulomatous inflammation	-
Fecal PCR	Positive (*Campylobacter*)	-
Repeat fecal PCR	Negative (*Campylobacter*)	-

**Table 2:** Blood investigations prior to and post-treatment for Whipple’s disease (after completion of one year of antibiotics). RBC: Red blood cell count; ESR: Erythrocyte sedimentation rate; CRP: C-reactive protein

Laboratory results	Pre-treatment	Post-treatment	Normal range
Hemoglobin	76	121	120-180 grams per liter (g/L)
RBC	2.87	3.61	3.5-5.5 x 10^12^/liter (L)
White blood cells	6.6	5.6	4-11 x 10^9^/liter (L)
ESR	44	16	3-9 millimeters per hour (mm/hr)
CRP	85	5	0-5 milligrams per liter (mg/L)
Albumin	27	42	35-50 grams per liter (g/L)
Weight	62.5 kg	83.5 kg	-

Upon completion of the antibiotic course, the patient demonstrated sustained improvement during clinical follow-up. Monitoring was recommended to ensure symptom recurrence did not occur, emphasizing the importance of clinical vigilance in managing complex cases such as the patient's.

In conclusion, the diagnostic journey of the patient illustrates the challenges and considerations in diagnosing and managing WD in the context of multiple comorbidities. Treatment based on histological findings proved effective in achieving symptom resolution, highlighting the necessity of tailored approaches in complex clinical scenarios.

## Discussion

WD is more prevalent in men than in women, with a male-to-female ratio of 2:3 [11]. Merely limited research has investigated its prevalence as well as incidence. Studies indicate a prevalence of 3/1,000,000 and an incidence of 0.1-0.6/1,000,000 new cases in Western populations. It is critical to investigate the differential diagnoses that arise among individuals with persistent constipation and loss of weight, and WD, despite its rarity, has emerged as a diagnostic possibility. In certain circumstances, WD might prove catastrophic if neglected [12,13]. TW can be detected in freshwater, soil, and marine sediments. It is present in 37% to 66% of sewage systems (influx) [14,15]. Farmers are particularly vulnerable to infections from polluted soil. TW is an uncommon disease, yet it affects 4% of the general population and 12% of undetected high-risk individuals, including sewer personnel.

Our case report includes an individual who had more classic signs but no skin hyperpigmentation unlike Batista et al. [16]. The woman ended up diagnosed with WD during the investigation to determine the cause of the clinical disease. In this case, HT, PH, PS, PAA, and LD were all involved, necessitating a more immediate diagnostic. Batista et al. [16] described a woman (58 years old) with skin excessive pigmentation, decreased weight (16% of body weight in three months) and appetite, upper abdomen pain, nausea, and diarrhea. OGD and colonoscopy were used for collecting biopsy specimens, which, combined with testing from the laboratory and microbiological examinations, resulted in the detection of WD. Our case report's individual age was lower than the reported study, and our findings were nearly identical to the above-mentioned published data, with the exception of the existence of nausea and skin pigmentation. Nunes et al. [17] described a 48-year-old individual who had a constitutional condition accompanied by an excessive bilateral pleural effusion. The small intestinal sample revealed a rod-like bacterial particle in the macrophage cytoplasm that was positive for PAS staining, while the PCR examination revealed TW DNA. The patient received a two-week endovenous antibacterial treatment of ceftriaxone 2 g per day, followed by a full year of oral trimethoprim 160 mg and sulfamethoxazole 800 mg twice a day (co-trimoxazole). She progressed well, with all symptoms resolved. The stated case report was determined to be consistent with ours except for pleural effusion, but our case had comorbidities with PS, PH, PAA, HT, and LD that were not present in the previously described case.

In accordance with our case, Najm et al. [18] indicated a 23-year-old male with WD-PH and suggested causes underlying the occurrence of PH in WD including the result of a cytokine-mediated proinflammatory state, direct infiltration of the pulmonary vasculature by TW, concurrent endocarditis or valvulopathy, or pulmonary emboli [19]. The study also found that antibiotics resulted in a considerable enhancement, indicating a major inflammatory impact. As a result, PAH-specific drugs might not be necessary as primary treatment in this setting. To our knowledge, no relationship between WD and PAA has yet been documented. Lagier et al. [20] determined that classical WD could result in subclinical HT and suggested comprehensive thyroid-stimulating hormone testing as routine in such a situation. The most plausible mechanism for the etiology could be TW infiltration into various tissues and fluids, resulting in thyroid tissue invasion and this scenario is verified by a decrease in biological differences following appropriate antibiotic therapy. The research team concluded that WD therapy could reverse TH and that thyroid supplementation was unnecessary to treat the individuals. Sarcoid-like tissue reactions are well-known to be an early manifestation of WD, and recognizing them may be useful in assisting with detection and therapy. According to most recent guidelines (2023), the diagnosis of WD with gastrointestinal predominant symptoms is made with upper gastrointestinal endoscopy and duodenal biopsies with at least five biopsies taken from the duodenum to avoid any sampling errors. Current diagnostic criteria require the detection of PAS-positive foamy macrophages in the histology of the small bowel biopsies. Once this is positive, WD is diagnosed. If this is negative, the diagnosis can be made by meeting two of the following criteria: (1) PAS staining showing foamy macrophages from extra-intestinal biopsied tissues; (2) PCR detection of TW or detection of specific 16S rRNA of the bacterium; (3) Immunohistochemistry staining with TW antibodies.

The duodenal biopsies of our patient revealed shortened and thickened villi. The lamina propria and villi were distended and packed with foamy macrophages. There was a positive strong granular pattern of PAS-D-resistant staining in these macrophages which was consistent with the presence of microorganisms. Overall appearances were in keeping with WD, thereby confirming the diagnosis of WD. Importantly, Ziehl-Neelsen staining was negative and there was no increase in intraepithelial lymphocytes or gastric metaplasia. PCR testing is an extremely sensitive and specific test and can be used for confirmation in inconclusive or suspicious cases [2]. It has also been used to monitor response to therapy [2]. Interestingly, the PCR testing for TW DNA was negative in our case. The histology report stated that the sensitivity of PCR is much reduced in FFPE (formalin fixed paraffin embedded) tissue and reported they had recently encountered a similar case in which PCR on FFPE material was negative and then positive on a repeat fresh sample. However, no existing data on the sensitivity of PCR testing on FFPE tissue versus fresh tissue samples could be found at the time of this report to support this claim.

This case also demonstrates that conditions such as PS, PAA, HT, and LD may occur as secondary diagnoses related to a primary diagnosis of WD, which could potentially be reversed with appropriate antibiotic treatment. No specific treatment is needed for the associated pathologies. Since the case presented demonstrated this association, it could be life-threatening if left untreated for WD.

## Conclusions

Our case report highlights the importance of using upper gastrointestinal endoscopy along with taking at least five duodenal biopsies, performing PAS staining, and conducting PCR detection for TW or 16S rRNA as first-line diagnostic tools. This approach is essential to prevent delays in diagnosing WD in patients who present with significant gastrointestinal symptoms. Additionally, the report stresses the need for ongoing follow-up, particularly in patients with WD, since recurrences are common and can occur even after completing a full course of antibiotic treatment.
